# Slice-illuminated optical projection tomography

**DOI:** 10.1364/OL.43.005555

**Published:** 2018-11-06

**Authors:** Samuel P. X. Davis, Laura Wisniewski, Sunil Kumar, Teresa Correia, Simon R. Arridge, Paul Frankel, James McGinty, Paul M. W. French

**Affiliations:** 1 Photonics Group, Department of Physics, Imperial College London, London, UK; 2 Division of Medicine, University College London, London, UK; 3 School of Biomedical Engineering and Imaging Sciences, King’s College London, London, UK

## Abstract

To improve the imaging performance of optical projection tomography (OPT) in live samples, we have explored a parallelized implementation of semi-confocal line illumination and detection to discriminate against scattered photons. Slice-illuminated OPT (sl-OPT) improves reconstruction quality in scattering samples by reducing interpixel crosstalk at the cost of increased acquisition time. For *in vivo* imaging, this can be ameliorated through the use of compressed sensing on angularly undersampled OPT data sets. Here, we demonstrate sl-OPT applied to 3D imaging of bead phantoms and live adult zebrafish.

There is increasing interest in performing *in situ* studies in live whole organisms for biomedical research. Fluorescence microscopy can provide *in vivo* molecular contrast, and techniques such as laser scanning confocal or multiphoton microscopy can produce three-dimensional (3D) images with sub-cellular resolution. However, as samples get larger (>1  mm), image acquisition times for whole live organisms become prohibitively long, resulting in significant phototoxicity and limiting potential study size. This challenge has led to the development of techniques such as optical projection tomography (OPT) [1], scanning laser optical tomography (SLOT) [2], and light sheet microscopy [3], which can be applied to “mesoscopic” (∼1–10  mm) samples, such as small animals and embryos.

Ideally these samples should be non-scattering. For *ex vivo* studies, this can be realized using chemical clearing techniques [1,4]. For *in vivo* studies, weakly scattering organisms can be studied, such as *D. melanogaster*, *C. elegans*, and *D. rerio* (zebrafish) embryos. Zebrafish embryos have become a popular model organism due to their rapid generation time and genetic accessibility. However, embryos are not appropriate for some disease studies, such as those requiring an adaptive immune system, a mature vasculature network, or observations over time scales exceeding a few days.

To address this need, we previously extended fluorescence OPT of live zebrafish embryos [5] to transgenic non-pigmented adult zebrafish [6,7] with the development of angularly multiplexed compressed-sensing OPT [8]. This combination of techniques enables longitudinal studies of tumor development with repeated imaging under anesthesia [9] and subsequent recovery [10]. However, although these non-pigmented fish are significantly more transparent than wild-type fish, they do present discernible optical scattering. Here, we aim to improve the image quality achievable by rejecting scattered light with semi-confocal illumination and detection.

OPT is the optical equivalent of x-ray computed tomography (CT), in which the 3D structure of a sample is reconstructed from a series of wide-field 2D projections acquired at different angles. Unlike light sheet microscopy and SLOT, scattering of excitation light does not degrade image quality in fluorescence OPT. However, all these techniques are impacted by scattering of fluorescence emission. In OPT, the recorded projection images are formed of ballistic and scattered photons. Optical scattering blurs the wide-field projections, degrading resolution and contrast.

For samples larger than the transport mean free path, scattering can be addressed with photon transport models, e.g., using diffuse optical tomography [11]. Weaker scattering can also be addressed computationally, e.g., [12,13], but it is desirable to reject scattered light optically where possible. For absorption OPT, the impact of scattering has been reduced using an ultrafast optical Kerr gate to form images with ballistic photons [14] or structured illumination to discriminate against scattered light [15]. However, ballistic fluorescence photons cannot be selected by time gating, due to the lifetime of the excited state, and the arithmetic operations involved in structured illumination reduce the available dynamic range [16]. To reduce the interpixel crosstalk caused by optical scattering without losing dynamic range, we have implemented parallelized semi-confocal imaging by selectively illuminating multiple slices of the sample and using synthetic multiple slit detection, which we describe as slice-illuminated OPT or “sl-OPT.” This approach directly removes photons that have been scattered laterally out of their incident trajectories, without using arithmetic operations.

A schematic of the sl-OPT setup is shown in Fig. 1

**Fig. 1.**
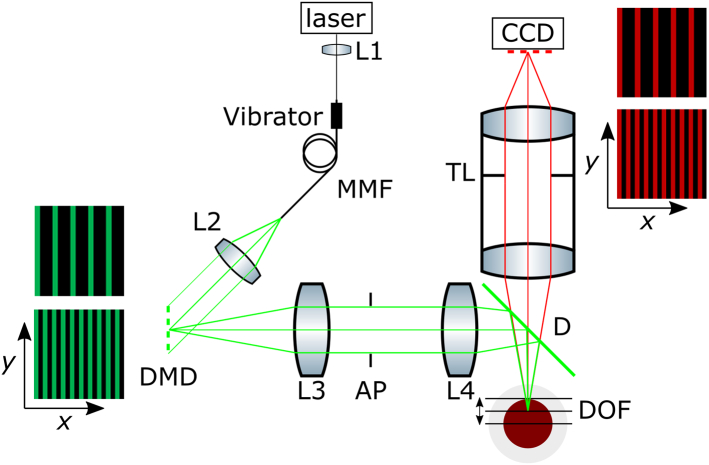
Experimental setup for sl-OPT showing the excitation and detection of multiple slices of radiation. Inset: representations of the DMD illumination patterns and CCD detection masks for a larger and smaller slice separation.

. Laser radiation at 561 nm (Jive, Cobolt) is coupled into a multimode optical fiber (MMF) by lens L1 (M43L02, Thorlabs, f=30  mm), collimated using lens L2 (AC f 100, Linos, f=100  mm) and directed to uniformly illuminate a digital micromirror device (DMD) with 1920×1080 pixels (DLP Lightcrafter 6500, TI) with laser speckle being averaged out by vibrating the MMF. The full field of the DMD is imaged onto the sample, via a dichroic filter D (Di03-R488/561, Semrock), using a telecentric imaging relay comprising achromatic lenses, L3 and L4 (AC508-300-A-ML, f=300  mm and AC508-500-A-ML, f=500  mm Thorlabs) with an adjustable aperture stop (AP) (SM2D25D, Thorlabs) in the Fourier plane. The excited fluorescence is imaged using a 0.5× magnification telecentric lens (TL) assembly with an adjustable aperture (TECHSPEC SilverTL, Edmund Optics), onto a charge-coupled device (CCD) camera (Clara, Andor) via an emission filter to block reflected excitation light (BA610IF, Olympus). The excitation and imaging systems are configured with optical axes parallel at the sample and perpendicular to the axis of rotation. Their focal planes are superimposed and the excitation and emission depths of focus set to 5 mm to cover at least the front half of the sample—giving a diffraction-limited resolution of 25.8 μm. The samples were mounted in fluorinated ethylene propylene (FEP) tubing (06406-72, Cole-Parmer) and suspended in a water-filled cuvette (704-003-50-10, Hellma Analytics) from a stepper motor (NM11AS-T4, Laser 2000 UK) with tip-tilt adjustment (M-TTN80, Newport Corp) and three-axis linear translation (M-423 and M-UMR12.63). Binary DMD patterns were composed in MATLAB and preloaded onto the DMD board.

The sample is illuminated with 25.3 μm thick parallel “slices” of excitation radiation by displaying a series of two-pixel-thick columns on the DMD. These excitation radiation slices are aligned parallel to the excitation optical axis and to the pixel columns of the CCD. A virtual mask is applied computationally to detect light only from the CCD pixel columns corresponding to the excitation slices. The positions of the virtual slits in the mask were determined by imaging a uniform dye phantom and using a best-fit linear mapping between the DMD and camera pixel positions.

For each projection image acquisition, the illumination pattern is scanned across the slice separation, one DMD pixel at a time, corresponding to 12.7 μm at the sample, thus realizing Nyquist sampling. Data from CCD pixels whose centers are more than 0.75 pixels away from the center of the virtual detection “slit” are discarded. This value was the minimum slit size that did not lead to striping artifacts caused by the different pitches of the DMD and CCD at the sample. No post-processing was required to correct misalignments between the excitation slices and virtual detection slits once the excitation light slices were aligned parallel to the optical axes and columns of the CCD.

Figure 2

**Fig. 2.**
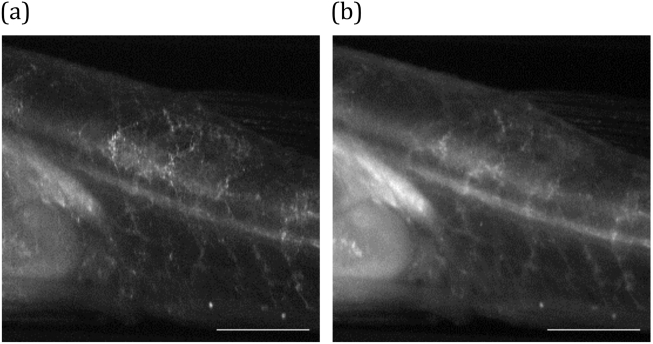
(a) sl-OPT projection image acquired with 100 μm slice separation and (b) pseudo-wide-field projection image of mCherryFP-labeled vasculature in an adult TraNac zebrafish. Scale bar 1 mm.

shows a sl-OPT projection image of vasculature in a terminally anaesthetized adult TraNac zebrafish (${mpv17}^{b18/b18}$ $mitfa^{w2/w2}$ $tg(kdrl\text{:}HRAS\text{-}m\mathrm{Cherry}{)}^{s896}$; $tg{\left(fabp10a\text{-}rtTA;\text{TETRE}:EGFP\text{-}kras\_G12V\right)}^{gz32}$)—where endothelial cells are labeled with mCherry fluorescent protein (mCherryFP)—at 56 days post fertilization [9]. For direct comparison, the corresponding pseudo-wide-field projection image (obtained by summing all images with no mask applied) is also shown. This shows the improved image quality provided by semi-confocal detection. To recover the sample volume, a set of slice-projections is taken at a number of equally spaced angles of sample rotation. The 3D tomographic image is then reconstructed utilizing the TwIST algorithm [17] implemented in MATLAB.

The slice separation should be optimized according to the scattering properties of the sample. As the separation is increased to reduce interpixel crosstalk, a point will be reached where there is no additional benefit to balance the increase in data acquisition time. To illustrate this, Fig. 3

**Fig. 3.**
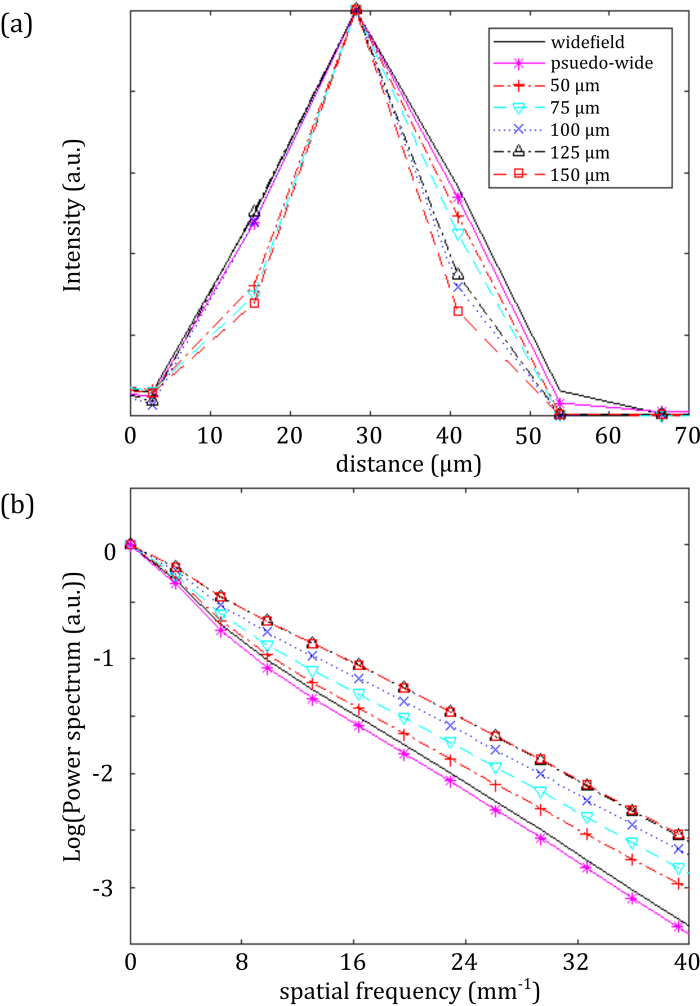
Amelioration of degradation of image resolution of 3D bead phantom as a function of slice separation: (a) line profile through a single bead and (b) normalized spatial frequency power spectrum of image for conventional OPT and sl-OPT with different slice separations.

shows data from a sl-OPT image acquisition of fluorescent beads (0.02 μm, Crimson 625/645 nm, Invitrogen) suspended in a 3D scattering phantom of 2% agarose, with scattering provided by a 0.05% by volume suspension of microspheres (Plain 1990 nm, PL-Microspheres). Wide-field projection images were recorded, followed by slice-illumination separated by 50 μm, 75 μm, 100 μm, 125 μm, and 150 μm in the sample, at 400 equally spaced angles, with a 500 ms integration time. Figure 3(a) shows a line profile through the maximum intensity projection of a single bead reconstructed from wide-field and sl-OPT data. To compare the imaging performance over the whole sample volume, their radial spatial frequency power spectra, averaged over the sphere, are plotted for the different slice separations in Fig. 3(b), with any DC offset in the output from TwIST being removed using a high-pass filter ($\sigma_{f}=0.54\;{\mathrm{mm}}^{-1}$). This illustrates how sl-OPT reduces the degradation to resolution compared to wide-field OPT, by suppressing low spatial frequency scattered light. This enhancement comes at the cost of increased acquisition time.

To quantify this, the ratios of the sl-OPT and wide-field spatial frequency power spectra, averaged over the top half of spatial frequencies supported by the modulation transfer function (i.e., $20–40\;{\mathrm{mm}}^{-1}$), were plotted as a function of the increase in acquisition time, as shown in Fig. 4

**Fig. 4.**
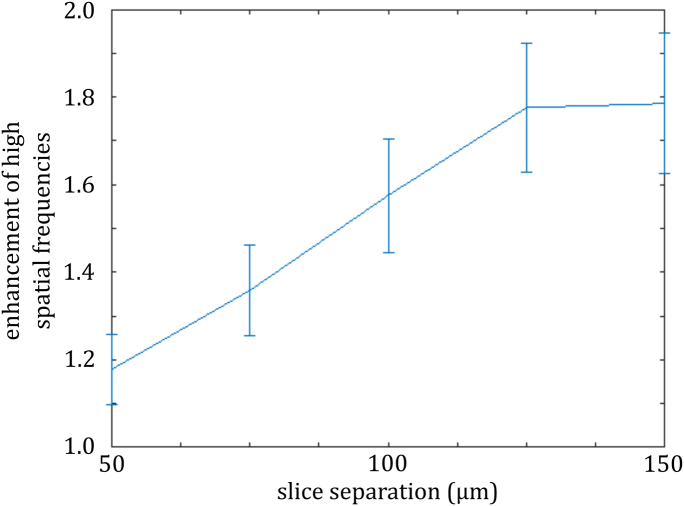
Enhancement of higher ($20–40\;{\mathrm{mm}}^{-1}$) spatial frequencies of reconstructed bead phantom images plotted as a function of slice separation (proportional to increased acquisition time for sl-OPT compared to wide-field OPT).

, which indicates that there is no benefit to image quality once the slice separation passes 125 μm. We note that Fig. 3 illustrates how the reconstructed image metrics based on pseudo-wide-field projections are similar to those resulting from true wide-field projections—indicating that the summing of readout noise when combining slice projection images does not significantly impact the reconstructed images.

To demonstrate sl-OPT *in vivo*, an adult zebrafish was imaged with projections acquired at 64 equally spaced angles, with 3 s CCD integration time. Figure 5

**Fig. 5.**
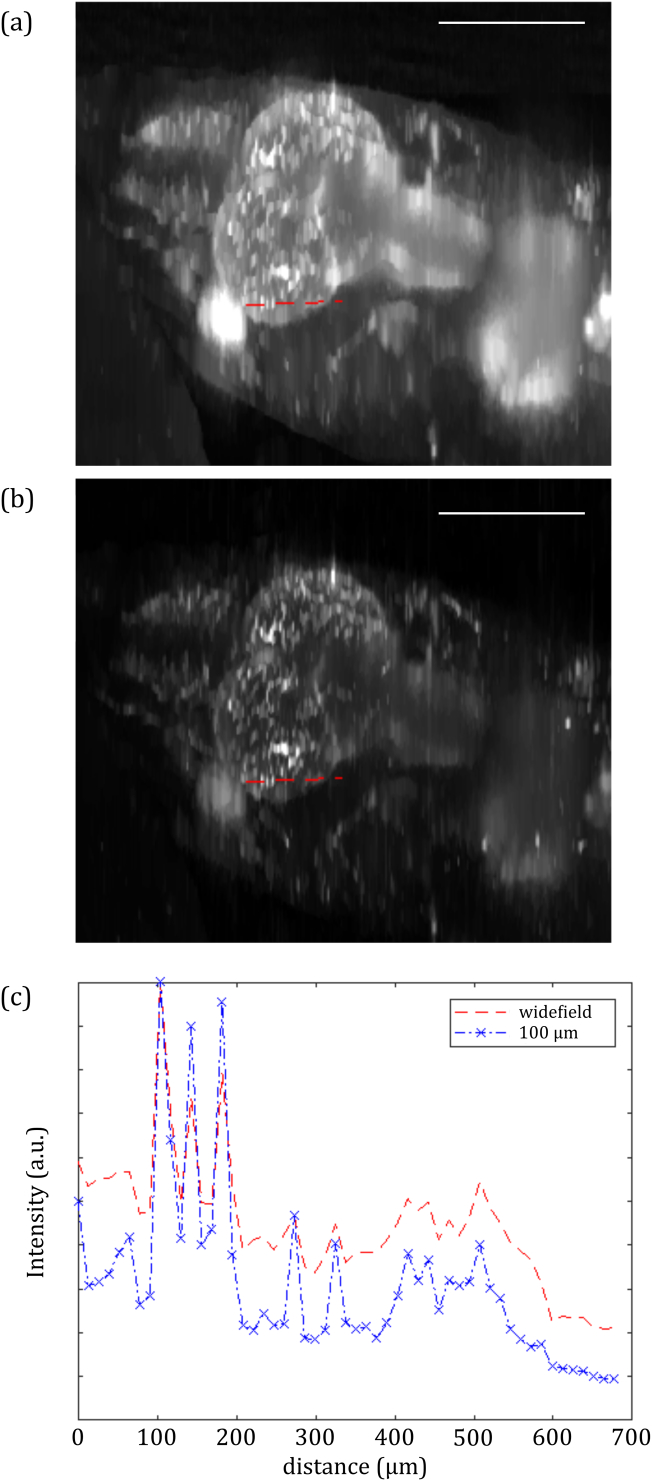
Maximum intensity projections of the head of a TraNac $tg{\left(kdrl\text{:}\mathrm{HRAS}-\text{mCherry}\right)}^{s896}$ zebrafish, cropped from a whole body reconstruction using (a) wide-field OPT, and (b) 100 μm separated sl-OPT. (c) Intensity profiles through indicated lines in (a) and (b). Scale bar 1 mm.

shows dorsal maximum intensity projections of a zebrafish reconstruction, cropped around the head. Figures 5(a) and 5(b) are reconstructed from pseudo-wide-field OPT and sl-OPT projections, and Fig. 5(c) presents line sections through a number of blood vessels, illustrating reduction in scattered background when using sl-OPT. Figure 6

**Fig. 6.**
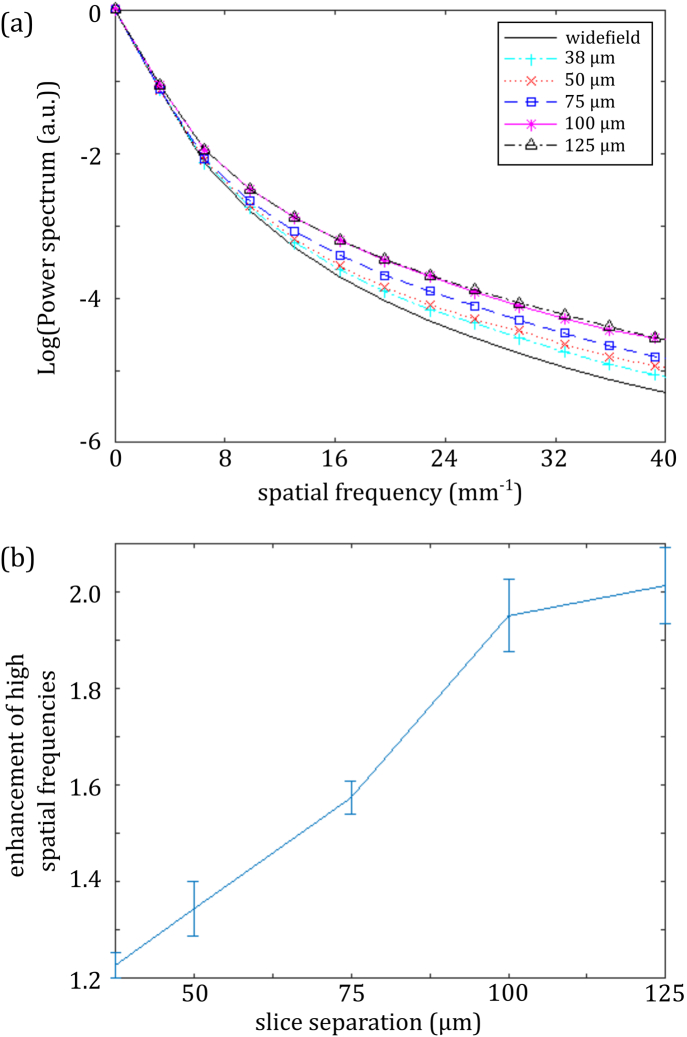
(a) Spatial frequency power spectra of reconstructed zebrafish volumes for pseudo-wide-field OPT, and sl-OPT data with different slice separations; (b) plot of enhancement of higher spatial frequencies ($20–40\;{\mathrm{mm}}^{-1}$) with slice separation (proportional to increased acquisition time for sl-OPT compared to wide-field OPT).

shows the imaging performance as a function of the slice projection separation, studied in the same way as for the 3D bead phantom. To minimize light dose and measurement time, data were acquired with slice-illumination separated by 75, 100, and 125 μm in the sample, with smaller separations being synthesized by summing together the appropriate raw projections. The enhancement of higher spatial frequencies is seen as the slice separation is increased—with a slice separation of 100 μm being optimal.

In conclusion, we have demonstrated that sl-OPT can enhance 3D imaging of weakly scattering samples by using semi-confocal line detection to suppress interpixel crosstalk due to scattered light. There is a tradeoff between the degree of image enhancement and the increase in data acquisition time, but since the technique can be combined with compressed sensing OPT, the total acquisition times can still be compatible with *in vivo* imaging of zebrafish under anesthesia. We note that sl-OPT does not enhance spatial resolution beyond what is achievable in cleared samples, unlike angular multiplexing [8] or focal scanning techniques [18]. However, these techniques do not reject scattered photons to ameliorate the image degradation in scattering samples due to interpixel crosstalk. Compared to post-processing approaches such as deconvolution and inverse scattering, sl-OPT does not require any assumptions of the optical properties of the sample. It could potentially be combined with such techniques.
